# Comparison of the effects of two different wound dressing materials on wound healing in mucosal defects created in diabetic and healthy rats

**DOI:** 10.1590/1678-7765-2026-0096

**Published:** 2026-08-14

**Authors:** Merve Yalçın, Vildan Bostancı, Özhan Karataş

**Affiliations:** 1 Sivas Cumhuriyet University Faculty of Dentistry Department of Periodontology Sivas Turkey Sivas Cumhuriyet University, Faculty of Dentistry, Department of Periodontology, Sivas, Turkey.; 2 Sivas Cumhuriyet University Faculty of Veterinary Medicine Division of Preclinical Sciences Sivas Turkey Sivas Cumhuriyet University, Faculty of Veterinary Medicine, Division of Preclinical Sciences, Department of Veterinary Pathology, Sivas, Turkey.

**Keywords:** Wound healing, Wound dressing, Diabetes mellitus, Rats

## Abstract

**Introduction:**

Diabetes mellitus impairs oral mucosal wound healing by prolonging inflammation and disrupting angiogenesis and growth factor regulation.

**Aim:**

To evaluate the effects of Ora-Aid (TBM Corporation, Gwangju, Republic of Korea) and cyanoacrylate (GluStitch, City of Industry, CA, USA) on palatal wound healing in diabetic and healthy rats.

**Methodology:**

A total of 146 male Wistar albino rats were used: 144 were divided into six groups: control (C), cyanoacrylate (CY), Ora-Aid (OA), diabetes (D), diabetes+cyanoacrylate (DCY), and diabetes+Ora-Aid (DOA) (n=24 each; 8 per time point), and 2 served as baseline controls. Diabetes was induced by intraperitoneal streptozotocin (STZ; Santa Cruz Biotechnology, Texas, USA) at 60 mg/kg. A 4-mm palatal excisional wound was created. Wound area was measured from photographs using ImageJ v1.54g (Wayne Rasband, National Institutes of Health, USA). Immunohistochemical analyses assessed vascular endothelial growth factor (VEGF), fibroblast growth factor (FGF), interleukin-10, and tumor necrosis factor-alpha. Data were analyzed using the Kruskal-Wallis test and Bonferroni-adjusted Mann-Whitney U tests. Statistical significance was evaluated based on adjusted p-values, with p<0.05 considered significant.

**Results:**

In healthy rats, wound areas after cyanoacrylate and Ora-Aid were similar to controls. In diabetic rats, both wound dressings reduced wound area, particularly on day 14 [median (IQR): D, 0.33 (0.82); DCY, 0.27 (0.71); DOA, 0.09 (0.09); D vs DCY, p=0.037; D vs DOA, p=0.002]. Immunohistochemical analyses showed that Ora-Aid more pronouncedly affected VEGF expression in healthy groups and FGF expression in diabetic groups, whereas cyanoacrylate showed time-dependent effects on growth factors and inflammatory markers.

**Conclusion:**

Ora-Aid and cyanoacrylate positively influenced oral mucosal wound healing under diabetic conditions. Ora-Aid more prominently supported the VEGF-related angiogenic response under healthy conditions and FGF-related fibroblastic activity under diabetic conditions, whereas cyanoacrylate showed time-dependent regulatory effects on growth factors and the inflammatory response.

## Graphical abstract

**Figure:**
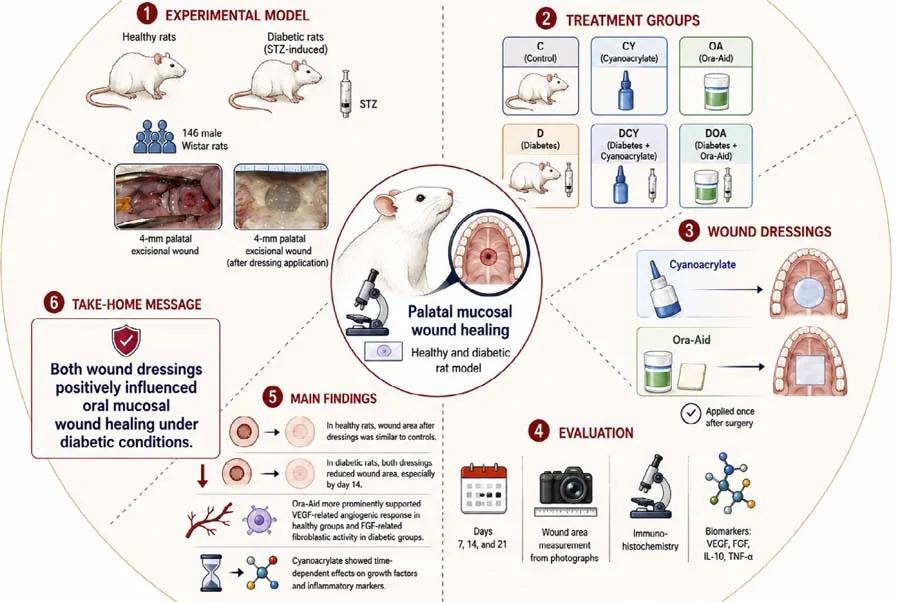

## Introduction

Wound healing is a dynamic and multi-stage biological process aimed at restoring tissue integrity.^1,2^ This process consists of four sequential phases: hemostasis, inflammation, proliferation, and remodeling.^3,^⁴ Various cells and mediators play roles in each phase.^1^ Among these mediators, vascular endothelial growth factor (VEGF) is one of the principal mediators of angiogenesis, playing an important role in endothelial cell proliferation and increased vascular permeability.⁵ Fibroblast growth factor (FGF) is another key growth factor involved in essential steps of wound repair, including granulation tissue formation, re-epithelialization, and tissue remodeling.^6-8^ Tumor necrosis factor-alpha (TNF-α) is one of the main proinflammatory cytokines that enhances the inflammatory response at the wound site.^9^ In contrast, interleukin-10 (IL-10) is an important anti-inflammatory cytokine that plays a role in limiting inflammation and supporting tissue repair.^10^

Diabetes mellitus is one of the major systemic diseases that negatively affects wound healing.^1 1,1 2^ Vascular damage resulting from chronic hyperglycemia, immune system dysfunction, and impaired inflammatory responses delay the healing process and increase the risk of complications.^11,13-15^ Although wounds in the oral mucosa normally tend to heal rapidly, this process can be significantly impaired in the presence of diabetes.^16^ In type 1 and type 2 diabetes mellitus models, oral mucosal wounds in diabetic animals have been shown to heal more slowly than normoglycemic controls.^17^ This condition may make wound management more critical, especially in clinically important areas such as the palatal region, which is frequently used as a donor site in periodontal and mucogingival surgery.^16-18^

In this context, wound dressing materials able to support oral mucosal wound healing and suitable for clinical use are of great importance. One such material, the n-butyl-2-cyanoacrylate tissue adhesive PeriAcryl® (GluStitch, City of Industry, CA, USA), possesses antimicrobial properties, rapid polymerization, and strong adhesion to mucosal surfaces.^19^ It has also been successfully used in oral surgical procedures.^20^ On the other hand, Ora-Aid^®^ (TBM Corporation, Gwangju, Republic of Korea) is a bioactive wound dressing with a hydrophilic polymer structure that provides a moist wound environment. It contains vitamin E (tocopherol), which may support the healing process.^2 1^

Under diabetic conditions, wound healing is significantly delayed due to increased free radical production and the resulting oxidative stress.^22^ The literature reports that vitamin E is a potent antioxidant that can reduce oxidative stress by inhibiting lipid peroxidation.^23^ Therefore, investigating the effect of Ora-Aid on wound healing, particularly under oxidative stress conditions triggered by diabetes, seems meaningful. Clinical studies have shown that Ora-Aid, used as an intraoral wound dressing, increases patient comfort after periodontal surgery, reduces postoperative pain and bleeding, and may support wound healing.^21,24^ However, the existing evidence is largely limited to clinical observations, and the biological effects of Ora-Aid on experimental mucosal wound healing, especially under diabetic conditions, have not been sufficiently elucidated.

Therefore, this study aimed to comparatively evaluate the effects of Ora-Aid and cyanoacrylate wound dressings on wound healing in palatal mucosal defects created in diabetic and healthy rats. This study hypothesized that applying Ora-Aid and cyanoacrylate would positively modulate wound healing under diabetic and healthy conditions by decreasing TNF-α expression and increasing VEGF, FGF, and IL-10 expression.

## Methodology

### Ethics Statement

The experimental protocol was approved by the Animal Experiments Ethics Committee of Cumhuriyet University Faculty of Medicine (Approval No: 18.01.2024-05). Prior to this approval, institutional permission was obtained from the Sivas Cumhuriyet University Experimental Animals Research and Application Center. All procedures were conducted in accordance with the ARRIVE guidelines and the relevant ethical regulations.

### Animals

A total of 146 healthy male Wistar albino rats aged 16 weeks and weighing 230–250 grams on average were used in this study. Only animals in good general health and no prior experimental procedures were included. Each experimental group was housed in separate cages under identical environmental conditions. All rats were maintained under a 12-hour light/12-hour dark photoperiod at 21 ± 1°C and 55–70% humidity, with ad libitum access to standard laboratory chow and water. Before the experimental procedures, the rats were acclimatized to their environment for 10 days to minimize stress levels.

The rats were assigned to experimental groups by a researcher not involved in the study, using a simple randomization method (drawing lots).

Animals in each group were further divided into three subgroups (n=8 per subgroup) to be sacrificed on days 7, 14, and 21 using the same simple randomization method (drawing lots). Additionally, two rats were designated as the day 0 baseline control group.

### Experimental Procedures – Surgical Procedure

To establish the experimental diabetes model, 72 rats received a single intraperitoneal injection of streptozotocin at a dose of 60 mg/kg. Plasma insulin levels were not evaluated in this study. Blood glucose levels were measured from tail vein blood samples using a glucometer (On Call Plus, ACON Biotech Co., Ltd., Hangzhou, China) three days after injection. Rats with blood glucose levels above 300 mg/dL were considered diabetic. Glucose levels of diabetic rats were monitored weekly throughout the experimental period.

Anesthesia was induced using Rompun (Bayer, Istanbul, Turkey) at 5 mg/kg intramuscularly (i.m.) and Ketalar (Pfizer, New York, USA) at 30 mg/kg i.m. A circular full-thickness excisional wound 4 mm in diameter was created in the midline of the palatal mucosa of all rats using a punch biopsy instrument.

Ora-Aid was prepared in approximately 6 × 6 mm dimensions to completely cover the wound area and minimally overlap the surrounding healthy tissue, and was then applied. Cyanoacrylate was applied locally as a thin layer covering the wound surface. Both materials were applied once at the time of surgery, and no additional carrier material was used.

### Sample Collection and Analysis

On days 0, 7, 14, and 21, the rats were sacrificed by intraperitoneal injection of 200 mg/kg pentothal sodium (Ekipental, Tümekip İlaç San., Istanbul, Turkey). For baseline evaluation, two rats were sacrificed on day 0; on days 7, 14, and 21, eight rats from each experimental group were analyzed. Their maxillae were then removed and prepared for analysis.

To evaluate wound areas, palatal regions were examined under a stereomicroscope at 30× magnification, and digital photographs were taken. Wound area measurements were performed on ImageJ v1 by an investigator blinded to group allocation.

Subsequently, tissue samples were excised from the hard palate mucosa, including the wound area and 1–2 mm of surrounding healthy tissue. This approach was chosen to enable the simultaneous evaluation of the wound margin and the adjacent healthy tissue and to ensure sampling standardization.

Immunohistochemical evaluation was performed by a pathologist blinded to the groups using a semi-quantitative scoring system in which the observed immunopositivity in the hard palate tissues was classified as absent (0), mild (1), moderate (2), or severe (3). However, no additional blinding procedure was applied during the allocation or statistical analysis stages.

### Immunohistochemical Tissue Preparation and Analysis

Hard palate tissue samples obtained during necropsy were fixed in 10% neutral formalin for 48 hours. After trimming, tissues were washed under running tap water for 8 hours. Samples underwent routine alcohol–xylene series and were embedded in paraffin blocks. Sections with 4 µm thickness were cut and placed on slides and deparaffinized in an incubator.

After washing with phosphate-buffered saline, endogenous peroxidase activity was blocked by incubating tissues in 3% hydrogen peroxide for 10 minutes. For antigen retrieval, tissues were treated with antigen retrieval solution at 500 watts for 2 × 5 minutes.

Following phosphate-buffered saline washing, tissues were incubated overnight (12 hours) at +4°C with the following primary antibodies:

- TNF-α (Affbiotech, Cincinnati, OH, USA; Catalog No: AF7014; dilution 1:200)
- IL-10 (Affbiotech, Cincinnati, OH, USA; Catalog No: DF6894; dilution 1:200)
- VEGF (Affbiotech, Cincinnati, OH, USA; Catalog No: AF5131; dilution 1:200)
- FGF (Affbiotech, Cincinnati, OH, USA; Catalog No: DF8946; dilution 1:200)

As secondary antibody, the large volume detection system: anti-polyvalent, HRP (Thermo Fisher; Catalog No: TP-125-HL) was used according to the manufacturer’s instructions.

DAB (3,3’-Diaminobenzidine) was used as the chromogen. After counterstaining with Mayer’s hematoxylin, slides were mounted with Entellan and examined under a light microscope.

### Statistical Analysis

The sample size was determined on G*Power (Version 3.1.9.4; Heinrich Heine University Düsseldorf, Düsseldorf, Germany) based on similar experimental studies.^25^ An a priori power analysis using one-way ANOVA with effect size f = 0.35, α = 0.05, target power = 0.90 (achieved power = 0.9092242), and six groups yielded a minimum required total sample size of 144.

Accordingly, 24 rats were planned for each group, distributed equally into 7-, 14-, and 21-day subgroups (n=8 each). Additionally, two rats were allocated for day 0 baseline histological/morphological data. Thus, a total of 146 rats were planned for use in this study (**Table 1**).

**Table 1:** Experimental groups, time points, and number of animals included in the study.

Group Code	Group Description	Time Points (Days)	Animals per Time Point (n)	Total (n)
**C**	Non-diabetic control	7, 14, 21	8	24
**CY**	Non-diabetic rats treated with cyanoacrylate	7, 14, 21	8	24
**OA**	Non-diabetic rats treated with Ora-Aid	7, 14, 21	8	24
**D**	Diabetic control	7, 14, 21	8	24
**DCY**	Diabetic rats treated with cyanoacrylate	7, 14, 21	8	24
**DOA**	Diabetic rats treated with Ora-Aid	7, 14, 21	8	24
**BC**	Baseline Control	0	2	2
**TOTAL**				**146**

Abbreviations: C, non-diabetic control; CY, non-diabetic rats treated with cyanoacrylate; OA, non-diabetic rats treated with Ora-Aid; D, diabetic control; DCY, diabetic rats treated with cyanoacrylate; DOA, diabetic rats treated with Ora-Aid; BC, baseline control. Note: Baseline control animals were sacrificed on day 0 for baseline histological assessment.

Although large effect sizes have been reported in the literature, a moderate-to-large effect size (f = 0.35) was conservatively selected since the diabetic model could increase biological variability and the multi-group design might result in more heterogeneous outcomes.

Statistical analyses were performed on IBM SPSS Statistics for Windows, version 20.0 (IBM Corp., Armonk, NY, USA). Due to the ordinal nature of the scoring system, non-parametric methods were preferred in this research. This study included six main experimental groups (C, CY, OA, D, DCY, and DOA), each further divided into independent subgroups corresponding to days 7, 14, and 21. Different rats were evaluated and sacrificed at each time point. Therefore, repeated measurements were not obtained from the same rats over time. Accordingly, repeated-measures statistical analyses were not applied. Descriptive statistics are shown as median and interquartile range (IQR). Moreover, 95% confidence intervals for medians were calculated using the Bootstrap method to provide estimates of precision for the wound area measurements. Comparisons between the six groups (C, CY, OA, D, DCY, and DOA) were performed using the Kruskal–Wallis test. To control for type I error inflation in multiple pairwise comparisons, Bonferroni-adjusted Mann–Whitney U tests were applied for post hoc analysis. Statistical significance was evaluated based on adjusted p-values, and all results were reported with exact p-values. A p-value<0.05 was considered statistically significant.

## Results

### Wound Area Findings

Wound healing progressed more slowly in the diabetes group than in the control group, and wound areas were significantly larger on day 14 (**Table 2** and **Table 3A**, p=0.049).

**Table 2:** Wound area (mm2) values measured at different time points in the experimental groups.

Group	Day 0	Day 7	Day 14	Day 21
**C**	6.66 (0.37) [6.47–6.85]	2.01 (3.40) [0.90–4.42]^ab^	0.36 (0.72) [0.13–0.98]^a^	0.19 (0.42) [0.03–0.62]^ab*^
**CY**	–	2.90 (2.39) [2.57–5.09]^ab^	1.22 (1.87) [0.57–2.43]^ab^	0.49 (0.82) [0.13–1.44]^ab*^
**OA**	–	1.51 (1.20) [1.06–2.35]^ab^	0.77 (0.70) [0.10–1.25]^ab*^	0.04 (0.12) [0.03–0.24]^ab*^
**D**	–	2.21 (0.50) [1.88–2.51]^a^	0.33 (0.82) [0.16–1.56]^b*^	0.32 (0.73) [0.03–1.02]^a*^
**DCY**	–	3.39 (1.14) [2.47–3.60]^a^	0.27 (0.71) [0.05–1.03]^a*^	0.05 (0.05) [0.03–0.26]^b*^
**DOA**	–	0.78 (1.20) [0.06–1.59]^b^	0.09 (0.09) [0.05–0.13]^a^	0.12 (0.50) [0.00–0.50]^ab^

Statistical analysis: Kruskal–Wallis test followed by Bonferroni-corrected Mann–Whitney U post hoc test. Abbreviations: C, non-diabetic control; CY, non-diabetic rats treated with cyanoacrylate; OA, non-diabetic rats treated with Ora-Aid; D, diabetic control; DCY, diabetic rats treated with cyanoacrylate; DOA, diabetic rats treated with Ora-Aid. Data are presented as median (IQR) [95% CI]. Different superscript lowercase letters (a, b) within the same column indicate statistically significant differences, whereas values sharing at least one common letter are not significantly different (p < 0.05). Accordingly, values indicated as “ab” are not statistically significantly different from values indicated as either “a” or “b.” The asterisk (*) indicates a statistically significant difference compared with day 7 within the same group (p < 0.05). 95% confidence intervals for medians were calculated using the Bootstrap method.

**Table 3:** Exact p values for overall and Bonferroni-adjusted pairwise comparisons of wound area. A) Bonferroni-adjusted pairwise comparisons performed between groups at each time point. B) Comparisons among independent time-point subgroups within each experimental condition and Bonferroni-adjusted pairwise comparisons

Time point	Comparison	Adjusted p value	Group	Overall comparison	Overall p value	Pairwise comparison	Adjusted p value
Day 7	C vs D	0.245					
Day 7	C vs CY	0.529	C	Day 0 vs Day 7 vs Day 14 vs Day 21	0.002	Day 21 vs Day 14	1.000
Day 7	C vs OA	0.916				Day 21 vs Day 7	0.014
Day 7	D vs DCY	0.775				Day 21 vs Day 0	0.017
Day 7	D vs DOA	0.002				Day 14 vs Day 7	0.186
Day 7	CY vs OA	0.115				Day 14 vs Day 0	0.093
Day 7	DCY vs DOA	0.001				Day 7 vs Day 0	1.000
Day 14	C vs D	0.049	CY	Day 7 vs Day 14 vs Day 21	0.002	Day 21 vs Day 14	0.359
Day 14	C vs CY	0.529				Day 21 vs Day 7	0.001
Day 14	C vs OA	0.958				Day 14 vs Day 7	0.162
Day 14	D vs DCY	0.037	OA	Day 7 vs Day 14 vs Day 21	0.004	Day 21 vs Day 14	1.000
Day 14	D vs DOA	0.002				Day 21 vs Day 7	0.006
Day 14	CY vs OA	0.529				Day 14 vs Day 7	0.024
Day 14	DCY vs DOA	0.183	D	Day 7 vs Day 14 vs Day 21	0.001	Day 21 vs Day 14	0.777
Day 21	C vs D	0.115				Day 21 vs Day 7	0.001
Day 21	C vs CY	0.495				Day 14 vs Day 7	0.047
Day 21	C vs OA	0.452	DCY	Day 7 vs Day 14 vs Day 21	0.001	Day 21 vs Day 14	1.000
Day 21	D vs DCY	0.007				Day 21 vs Day 7	0.002
Day 21	D vs DOA	0.064				Day 14 vs Day 7	0.020
Day 21	CY vs OA	0.325	DOA	Day 7 vs Day 14 vs Day 21	0.057	–	–
Day 21	DCY vs DOA	0.668					

Statistical analysis: Kruskal–Wallis test followed by Bonferroni-corrected Mann–Whitney U post hoc test.

Abbreviations: C, non-diabetic control; CY, non-diabetic rats treated with cyanoacrylate; OA, non-diabetic rats treated with Ora-Aid; D, diabetic control; DCY, diabetic rats treated with cyanoacrylate; DOA, diabetic rats treated with Ora-Aid.

In diabetic rats, both cyanoacrylate and Ora-Aid applications significantly reduced the wound area at specific time points when compared to the diabetic control group (**Table 2** and **Table 3A**, p<0.05).

On day 7, the wound area in the diabetes + Ora-Aid group was significantly smaller than that in the diabetes + cyanoacrylate group (**Table 2** and Table 3A, p=0.001). However, no statistically significant difference in wound area was detected between the two treatment groups on days 14 and 21 (**Table 2** and **Table 3A**, p>0.05).

The time-dependent changes in wound area between the experimental groups are shown in **Figure 1**. The macroscopic healing process of the palatal wounds over time is shown in stereomicroscopic images in Supplementary Figure 1. Exact p-values for the overall comparisons and Bonferroni-adjusted pairwise comparisons of wound area are shown in **Tables 3A and 3B**.

**Figure 1:**
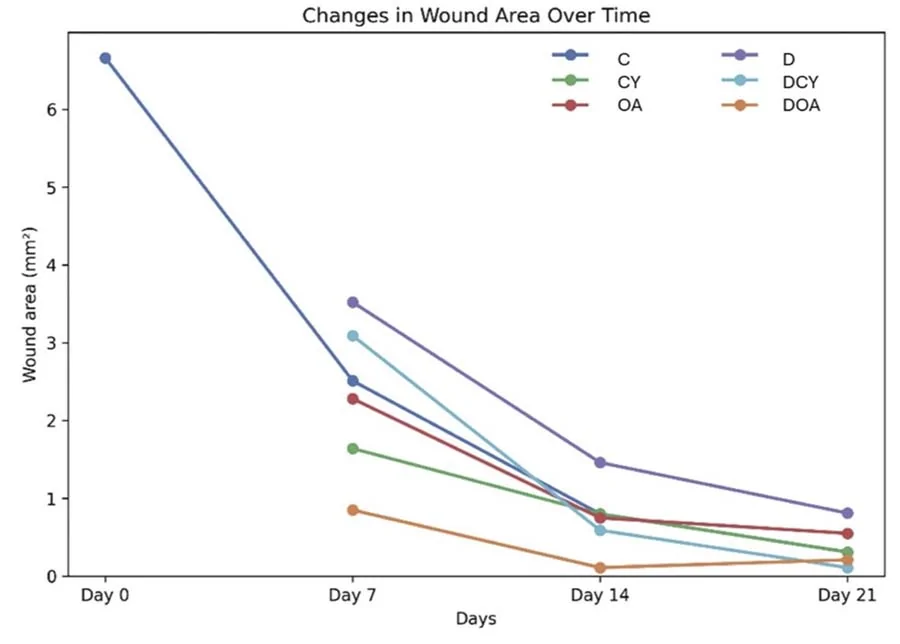
Changes in wound area (mm2) over time in the experimental groups. Day 0 represents the baseline wound area in the control group. Corresponding descriptive statistics and statistical comparisons are shown in Tables 2, 3A, and 3B. Abbreviations: C, Control; D, Diabetes; CY, Cyanoacrylate; OA, Ora-Aid; DCY, diabetic rats treated with cyanoacrylate; DOA, diabetic rats treated with Ora-Aid.

### Immunohistochemical Findings

The data are shown as median and IQR, obtained from a semi-quantitative immunohistochemical evaluation by a pathologist blinded to group assignments.

When VEGF immunopositivity was evaluated, no marked VEGF immunopositivity was observed in the C0 and C7 groups. Mild expression was detected in the C14, D7, D14, D21, CY7, CY21, DCY7, DCY14, DOA7, DOA14, and DOA21 groups. Moderate expression was observed in the C21 and CY14 groups, whereas strong expression was detected in the OA7, OA14, OA21, and DCY21 groups (**Figure 2**).

**Figure 2:**
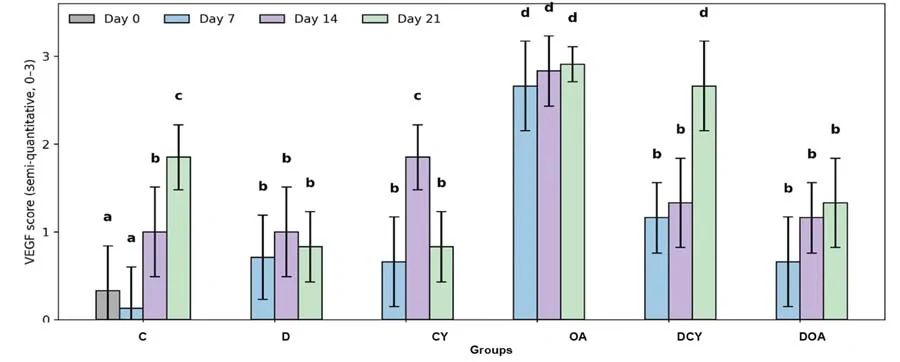
Semi-quantitative distribution of VEGF immunohistochemical expression according to groups and time points. Different letters (a–d) indicate statistically significant differences between groups and time points based on immunohistochemical evaluation. Values sharing the same letter are not significantly different (p<0.05). Abbreviations: C, Control; D, ‘Diabetes; CY, cyanoacrylate; OA, Ora-Aid; DCY, diabetic rats treated with cyanoacrylate; DOA, diabetic rats treated with Ora-Aid.

Representative immunohistochemical staining patterns of VEGF in palatal mucosal tissues are shown (**Figure 3**). The median (IQR) values of VEGF scores and the exact p-values for comparisons between time points within groups are shown in the relevant table (**Table 4**).

**Figure 3:**
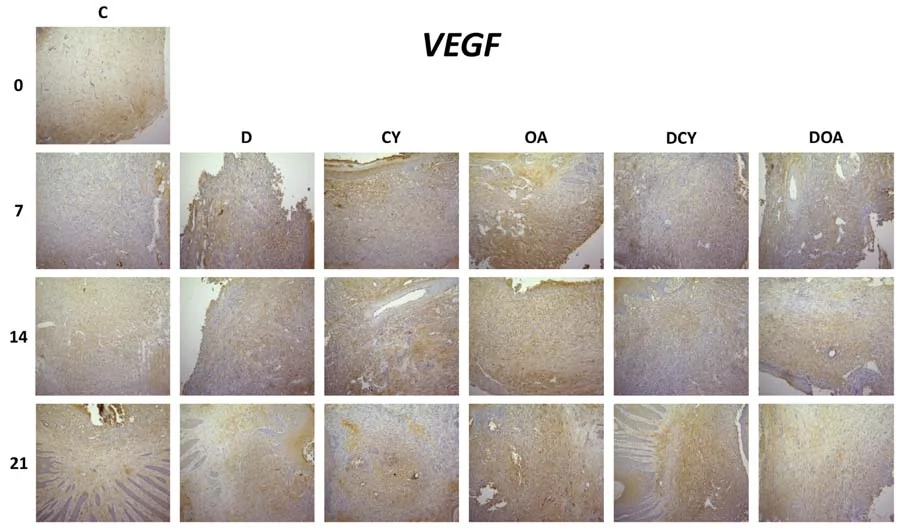
Immunohistochemical appearance of VEGF expression in palatal mucosal tissues on days 0, 7, 14, and 21. Brown staining indicates positive immunoreactivity (×10 magnification). Abbreviations: C, Control; D, Diabetes; CY, cyanoacrylate; OA, Ora-Aid; DCY, diabetic rats treated with cyanoacrylate; DOA, diabetic rats treated with Ora-Aid.

**Table 4:** Semi-quantitative VEGF immunohistochemical scores at different time points in the experimental groups.

Groups	Day 7 Median (IQR)	Day 14 Median (IQR)	Day 21 Median (IQR)	p-value*
**Group C**	0.00 (0.00–0.25)	1.00 (0.75–1.00)	2.00 (2.00–2.00)	< 0.001
**Group D**	1.00 (0.75–1.00)	1.00 (1.00–1.00)	2.00 (2.00–2.00)	< 0.001
**Group CY**	0.50 (0.00–1.00)	2.00 (2.00–2.00)	1.00 (1.00–1.00)	< 0.001
**Group OA**	3.00 (2.75–3.00)	3.00 (2.75–3.00)	3.00 (2.75–3.00)	< 0.001
**Group DCY**	1.00 (1.00–1.25)	1.00 (1.00–1.25)	3.00 (2.00–3.00)	< 0.001
**Group DOA**	1.00 (0.75–1.00)	1.00 (1.00–1.25)	2.00 (1.75–2.00)	< 0.001

Data are presented as median (IQR). The p-value represents the exact p-value of the Kruskal–Wallis test for comparisons across time points within each group. For a complementary statistical interpretation of the immunohistochemical scores, please also refer to Figure 2. Abbreviations: C, non-diabetic control; CY, non-diabetic rats treated with cyanoacrylate; OA, non-diabetic rats treated with Ora-Aid; D, diabetic control; DCY, diabetic rats treated with cyanoacrylate; DOA, diabetic rats treated with Ora-Aid.

When FGF immunopositivity was evaluated, FGF expression was found at a very low level in the C0, D21, and DCY7 groups. Mild expression was observed in the C7, D7, D14, CY14, CY21, OA7, DCY14, and DCY21 groups. Moderate FGF immunopositivity was detected in the C21, OA14, OA21, DOA7, DOA14, and DOA21 groups, whereas strong expression was identified in the C14 and CY7 groups (**Figure 4**).

**Figure 4:**
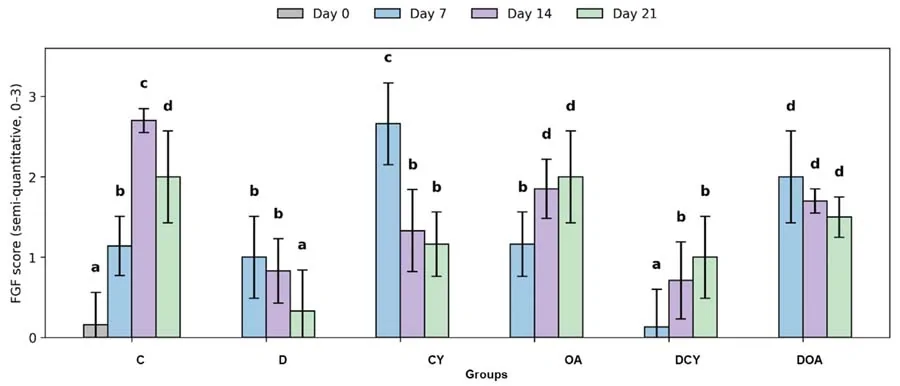
Semi-quantitative distribution of FGF immunohistochemical expression according to groups and time points. Different letters (a–d) indicate statistically significant differences between groups and time points based on immunohistochemical evaluation. Values sharing the same letter are not significantly different (p<0.05). Abbreviations: C, Control; D, Diabetes; CY, cyanoacrylate; OA, Ora-Aid; DCY, diabetic rats treated with cyanoacrylate; DOA, diabetic rats treated with Ora-Aid.

Representative immunohistochemical staining patterns of FGF in palatal mucosal tissues are shown (**Figure 5**). The median (IQR) values of FGF scores and the exact p-values for comparisons between time points within groups are shown in the relevant table (**Table 5**).

**Figure 5:**
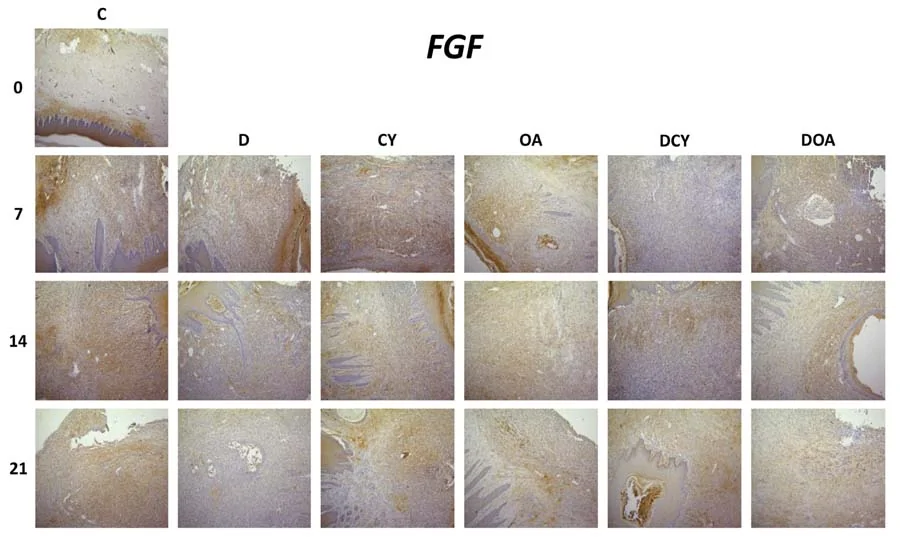
Immunohistochemical appearance of FGF expression in palatal mucosal tissues on days 0, 7, 14, and 21. Brown staining indicates positive immunoreactivity (×10 magnification). Abbreviations: C, Control; D, Diabetes; CY, cyanoacrylate; OA, Ora-Aid; DCY, diabetic rats treated with cyanoacrylate; DOA, diabetic rats treated with Ora-Aid.

**Table 5:** Semi-quantitative FGF immunohistochemical scores at different time points in the experimental groups.

Groups	Day 7 Median (IQR)	Day 14 Median (IQR)	Day 21 Median (IQR)	p-value*
**Group C**	1.00 (1.00–1.00)	3.00 (2.75–3.00)	2.00 (2.00–2.00)	< 0.001
**Group D**	1.00 (1.00–1.00)	1.00 (0.75–1.00)	0.00 (0.00–0.25)	< 0.001
**Group CY**	3.00 (2.75–3.00)	1.00 (1.00–1.25)	1.00 (1.00–1.00)	< 0.001
**Group OA**	1.00 (1.00–1.25)	2.00 (1.75–2.00)	2.00 (2.00–2.00)	< 0.001
**Group DCY**	0.00 (0.00–0.25)	1.00 (0.75–1.00)	1.00 (1.00–1.00)	< 0.001
**Group DOA**	2.00 (1.75–2.25)	2.00 (1.75–2.00)	2.00 (1.00–2.00)	< 0.001

Data are presented as median (IQR). The p-value represents the exact p-value of the Kruskal–Wallis test for comparisons across time points within each group. For a complementary statistical interpretation of the immunohistochemical scores, please also refer to Figure 4. Abbreviations: C, non-diabetic control; CY, non-diabetic rats treated with cyanoacrylate; OA, non-diabetic rats treated with Ora-Aid; D, diabetic control; DCY, diabetic rats treated with cyanoacrylate; DOA, diabetic rats treated with Ora-Aid.

When IL-10 immunopositivity was evaluated, IL-10 expression was found at a very low level in the C0, D21, CY21, OA7, OA14, OA21, DCY7, DCY14, DOA14, and DOA21 groups. Mild expression was observed in the C7, D14, CY7, CY14, DCY21, and DOA7 groups. Moderate IL-10 immunopositivity was detected in the C21 and D7 groups, whereas strong expression was identified in the C14 group (**Figure 6**).

**Figure 6:**
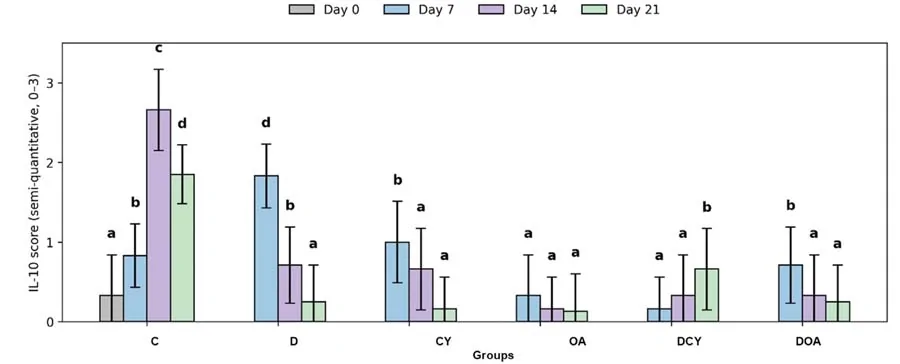
Semi-quantitative distribution of IL-10 immunohistochemical expression according to groups and time points. Different letters (a–d) indicate statistically significant differences between groups and time points based on immunohistochemical evaluation. Values sharing the same letter are not significantly different (p<0.05). Abbreviations: C, Control; D, Diabetes; CY, cyanoacrylate; OA, Ora-Aid; DCY, diabetic rats treated with cyanoacrylate; DOA, diabetic rats treated with Ora-Aid

Representative immunohistochemical staining patterns of IL-10 in palatal mucosal tissues are shown (**Figure 7**). The median (IQR) values of IL-10 scores and the exact p-values for comparisons between time points within groups are shown in the relevant table (**Table 6**).

**Figure 7:**
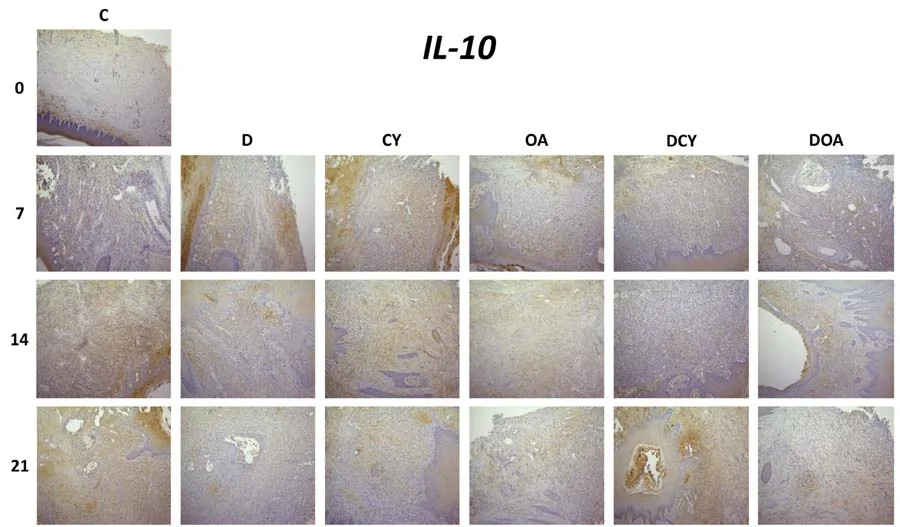
Immunohistochemical appearance of IL-10 expression in palatal mucosal tissues on days 0, 7, 14, and 21. Brown staining indicates positive immunoreactivity (×10 magnification). Abbreviations: C, Control; D, Diabetes; CY, cyanoacrylate; OA, Ora-Aid; DCY, diabetic rats treated with cyanoacrylate; DOA, diabetic rats treated with Ora-Aid.

**Table 6:** Semi-quantitative IL-10 immunohistochemical scores at different time points in the experimental groups.

Groups	Day 7 Median (IQR)	Day 14 Median (IQR)	Day 21 Median (IQR)	p-value*
**Group C**	1.00 (1.00–1.00)	3.00 (2.75–3.00)	2.00 (2.00–2.00)	< 0.001
**Group D**	2.00 (2.00–2.00)	1.00 (0.00–1.00)	0.00 (0.00–0.00)	< 0.001
**Group CY**	1.00 (1.00–1.25)	1.00 (1.00–1.00)	0.00 (0.00–0.00)	< 0.001
**Group OA**	0.50 (0.00–1.00)	0.00 (0.00–0.25)	0.00 (0.00–0.00)	< 0.001
**Group DCY**	0.00 (0.00–0.00)	0.00 (0.00–0.25)	0.00 (0.00–1.25)	< 0.001
**Group DOA**	1.00 (0.75–1.25)	0.00 (0.00–0.25)	0.00 (0.00–0.00)	< 0.001

Data are presented as median (IQR). The p-value represents the exact p-value of the Kruskal–Wallis test for comparisons across time points within each group. For a complementary statistical interpretation of the immunohistochemical scores, please also refer to Figure 6. Abbreviations: C, non-diabetic control; CY, non-diabetic rats treated with cyanoacrylate; OA, non-diabetic rats treated with Ora-Aid; D, diabetic control; DCY, diabetic rats treated with cyanoacrylate; DOA, diabetic rats treated with Ora-Aid.

When TNF-α expression was evaluated, strong immunopositivity was observed in the C0, C7, and C14 groups. Moderate expression was detected in the C21, D7, DOA7, and DOA14 groups. Mild immunopositivity was observed in the D14, D21, CY7, CY14, CY21, OA7, OA14, OA21, DCY7, and DCY21 groups. In contrast, TNF-α expression was found at a very low level in the DCY14 and DOA21 groups (**Figure 8**).

**Figure 8:**
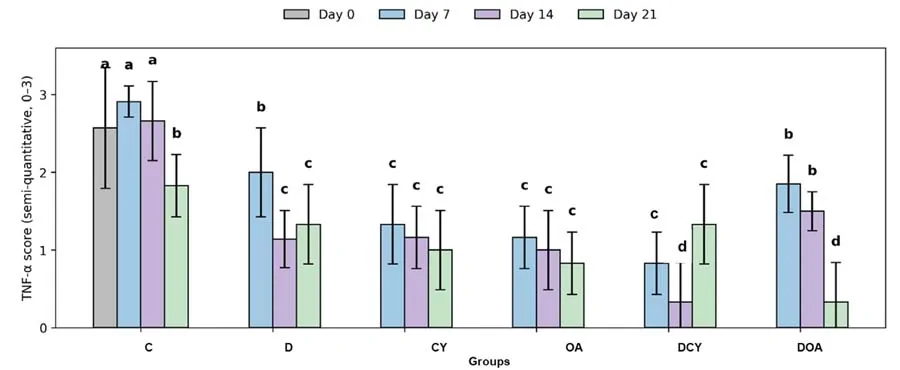
Semi-quantitative distribution of TNF-α immunohistochemical expression according to groups and time points. Different letters (a–d) indicate statistically significant differences between groups and time points based on immunohistochemical evaluation. Values sharing the same letter are not significantly different (p<0.05). Abbreviations: C, Control; D, Diabetes; CY, cyanoacrylate; OA, Ora-Aid; DCY, diabetic rats treated with cyanoacrylate; DOA, diabetic rats treated with Ora-Aid.

Representative immunohistochemical staining patterns of TNF-α in palatal mucosal tissues are shown (**Figure 9**). The median (IQR) values of TNF-α scores and the exact p-values for comparisons between time points within groups are shown in the relevant table (**Table 7**).

**Figure 9:**
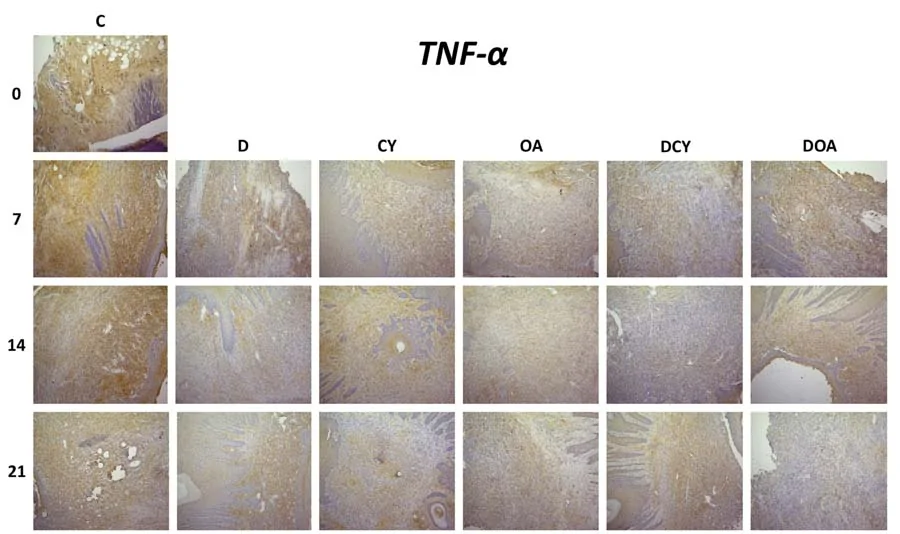
Immunohistochemical appearance of TNF-α expression in palatal mucosal tissues on days 0, 7, 14, and 21. Brown staining indicates positive immunoreactivity (×10 magnification). Abbreviations: C, Control; D, Diabetes; CY, cyanoacrylate; OA, Ora-Aid; DCY, diabetic rats treated with cyanoacrylate; DOA, diabetic rats treated with Ora-Aid.

**Table 7:** Semi-quantitative TNF-α immunohistochemical scores at different time points in the experimental groups.

Groups	Day 7 Median (IQR)	Day 14 Median (IQR)	Day 21 Median (IQR)	p-value*
**Group C**	3.00 (3.00–3.00)	3.00 (2.75–3.00)	2.00 (2.00–2.00)	0.002
**Group D**	2.00 (2.00–2.00)	1.00 (1.00–1.25)	1.00 (1.00–2.00)	0.012
**Group CY**	1.00 (1.00–2.00)	1.00 (1.00–2.00)	1.00 (1.00–1.00)	0.015
**Group OA**	1.00 (1.00–1.25)	1.00 (1.00–1.00)	1.00 (0.75–1.00)	0.008
**Group DCY**	1.00 (0.75–1.00)	0.00 (0.00–0.25)	1.00 (1.00–1.25)	0.004
**Group DOA**	2.00 (1.75–2.00)	1.50 (1.00–2.00)	0.00 (0.00–0.25)	0.001

Data are presented as median (IQR). The p-value represents the exact p-value of the Kruskal–Wallis test for comparisons across time points within each group. For a complementary statistical interpretation of the immunohistochemical scores, please also refer to Figure 8. Abbreviations: C, non-diabetic control; CY, non-diabetic rats treated with cyanoacrylate; OA, non-diabetic rats treated with Ora-Aid; D, diabetic control; DCY, diabetic rats treated with cyanoacrylate; DOA, diabetic rats treated with Ora-Aid.

## Discussion

In this study, the effects of Ora-Aid and cyanoacrylate wound dressings on oral mucosal wound healing were comparatively evaluated at macroscopic and immunohistochemical levels in healthy and diabetic rat models. The findings revealed that the effects of wound dressing materials on the healing process differ depending on systemic condition and time. It was particularly observed that both materials significantly affected wound area and immune marker profiles under diabetic conditions (whereas this effect remained limited in healthy groups). In this section, the obtained results will be discussed in light of the literature via key markers related to angiogenesis, fibroblast activity, and inflammatory response.

The macroscopic findings showed that Ora-Aid and cyanoacrylate applications provided a time-dependent significant reduction in wound area in diabetic groups, having no notable effect on wound area dimensions in healthy groups. This suggests that the efficacy of wound dressing materials in oral mucosal wounds may be closely related to systemic factors. In the literature, Kang, et al. reported that, in a healthy rabbit model, excisional and chemical burn wounds closed more rapidly in Ora-Aid-treated groups than in the control group.^28^ Similarly, Albayrak, et al.^27^ (2026) showed that a wound dressing composed of a bioactive material combination supported clinical healing in an experimental oral ulcer model. Furthermore, various topical materials have been reported to support clinical wound healing in palatal wound models, reduce the unhealed area, or accelerate wound closure.^25,26,29^ However, the animal model, wound type, physical structure of the material, evaluation method, and systemic conditions differ across most of these studies. Therefore, direct one-to-one comparison with the findings in this study should be made with caution. Moreover, the limited number of studies in which Ora-Aid and cyanoacrylate have been comparatively evaluated based on macroscopic wound area in the same palatal mucosal defect model under both healthy and diabetic conditions increases the contribution of this study to the literature.

VEGF is a fundamental growth factor that plays a role in various stages of the wound healing process, including angiogenesis, epithelization, and collagen deposition.⁵ In our study, VEGF expression was observed to be higher in the healthy group treated with Ora-Aid than in the control and other treatment groups, and VEGF expression in the healthy cyanoacrylate group increased when compared to the control group on day 14. In Gül, et al., who evaluated secondary wound healing in the palatal region, VEGF expression in the control group was reported to be lower on days 7, 14, and 21 than in groups treated with topical agents, which is consistent with our findings.^30^ Similarly, Assar, et al., examining the effects of licorice root extract on wound healing in a rat model, showed that VEGF gene expression was higher in treated groups than in the control group and that topical application provided a more pronounced effect than oral administration.^31^ Although the agents in the aforementioned studies differ, their findings are comparable with the ones in our study as they show the effect of topical applications on VEGF expression. In diabetic groups, VEGF expression generally remained at low levels. However, a notable increase was observed in the diabetes + cyanoacrylate group on day 21. These findings suggest that Ora-Aid application may support the VEGF-mediated angiogenic response under healthy conditions, but this effect may be limited in the diabetic environment. In contrast, cyanoacrylate application seemed to be effective on VEGF expression in the early period in healthy groups and in the later period in diabetic groups.

FGF is an important growth factor that plays a role in various stages of wound healing, such as fibroblast activity, re-epithelialization, and angiogenesis. It has been reported that FGF expression decreases in the presence of diabetes due to oxidative stress and hyperglycemia, which adversely affects fibroblast activity and tissue regeneration.^32^ Brizeno, et al.^17^ (2016) showed that FGF-2 expression levels during healing of oral mucosal ulcers in diabetic rats were lower than in healthy groups. Similarly, in our study, FGF expression in the control group was found to be higher than in the diabetic group on days 14 and 21. Additionally, cyanoacrylate application in the healthy group was observed to increase FGF expression on day 7. When diabetic groups (D, DCY, and DOA) were evaluated, Ora-Aid application increased FGF immunopositivity when compared to the diabetes group at all time points, whereas cyanoacrylate application increased it particularly on day 21. FGF levels in the diabetes + Ora-Aid group were found to be higher than those in the diabetes + cyanoacrylate group at all time points. These findings suggest that Ora-Aid may show a more pronounced effect in supporting the fibroblast response under diabetic conditions, whereas cyanoacrylate may be effective in the early period in healthy tissues and in the later period in diabetic tissues.

TNF-α is one of the key pro-inflammatory cytokines that plays a role in the inflammatory phase of wound healing, and is expected to increase in the early period.^3 3^ In the literature, studies have reported that TNF-α expression increases in diabetic models when compared to control groups. Brizeno, et al. reported higher TNF-α expression in the epithelium during healing of oral mucosal ulcers in diabetic rats.^1^⁷ Similarly, Hedayatyanfard, et al.^3^⁴ (2020) reported that TNF-α levels increased on day 14 in a wound model created in diabetic rats when compared to the control group. In contrast, in our study, TNF-α expression remained at lower levels in the diabetes group, suggesting that diabetes may modulate the inflammatory response and suppress the early inflammatory phase. Furthermore, cyanoacrylate and Ora-Aid applications maintained TNF-α levels at lower levels than in the control group at all time points, contributing to the regulation of inflammation. In diabetic conditions, the suppression of TNF-α expression by cyanoacrylate in the early period and by Ora-Aid in the later period suggests that the effects of these materials on the inflammatory response may vary depending on time.

IL-10 is an anti-inflammatory cytokine that plays a critical role in the resolution of inflammation and the initiation of tissue repair.^3^⁵ In our study, IL-10 levels were high in the control group on days 14 and 21, whereas they generally remained at lower levels in diabetic groups. This finding suggests that the inflammatory process may be inadequately resolved in the presence of diabetes, which may contribute to prolonged healing. On days 14 and 21, IL-10 levels in healthy groups treated with cyanoacrylate and Ora-Aid were significantly lower than in the control group. When diabetic groups were evaluated, IL-10 levels in the diabetes group on days 7 and 14 were observed to be higher than in treated diabetic groups. However, on day 21, while the diabetes group and the diabetes + Ora-Aid group showed similar IL-10 levels, significantly higher IL-10 expression was detected in the diabetes + cyanoacrylate group. These findings suggest that cyanoacrylate application may contribute more effectively to the resolution of inflammation in the later period under diabetic conditions. Aravinthan, et al.^36^ (2018), evaluating the effects of collagen-based materials in a skin incision model created on the lateral regions of rats, found IL-10 levels to be higher in treatment groups compared to the control group. These differences, which seem inconsistent with the literature, may be attributable to differences in the wound model, the biological properties of the applied material, and the evaluation time points.

In this context, the low IL-10 levels in treatment groups can be explained by two possible mechanisms: insufficient activation of the inflammatory response, or the applied materials accelerating wound healing so that the tissue transitions to the proliferation and remodeling phase earlier, eliminating the need for IL-10 to remain at high levels for an extended period. In future studies, evaluating IL-10 levels with other healing parameters such as collagen deposition and scar quality will contribute to a clearer interpretation of these findings.

The initial hypothesis of this study was that Ora-Aid and cyanoacrylate applications would positively modulate wound healing under healthy and diabetic conditions, reducing TNF-α expression while increasing VEGF, FGF, and IL-10 expression. The findings partially confirmed this hypothesis. Macroscopic healing findings along with VEGF and FGF results supported the hypothesis, whereas TNF-α and especially IL-10 findings showed neither change in the expected direction nor consistency across all groups and time points. Therefore, the study hypothesis was partially, rather than entirely, supported.

From a clinical perspective, this study provides an experimental foundation, particularly regarding the supportive use of wound dressing materials in delayed oral mucosal wound healing associated with diabetes. The obtained findings suggest that Ora-Aid and cyanoacrylate may provide potential benefits in wound management following periodontal, mucogingival, and other oral surgical procedures. These materials may offer noteworthy options, especially for approaches to support the healing process in diabetic patients. Although studies in the literature have examined the effects of different wound dressing materials on oral mucosal wound healing, the number of studies in which Ora-Aid and cyanoacrylate have been comparatively evaluated in a palatal mucosal defect model under both healthy and diabetic conditions (with their effects examined jointly by both growth factors and inflammatory markers) is limited. Thus, this study contributes to an important gap in the literature regarding the biological effects of wound dressing materials according to systemic conditions.

Certain limitations of this study should be taken into consideration. First, this research being conducted on an experimental rat model limits the direct applicability of its findings to human oral mucosa. Additionally, the 21-day follow-up period prohibited the evaluation of late-stage tissue remodeling and long-term healing dynamics. The semi-quantitative evaluation of immunohistochemical findings is another important limitation of the study: while this approach is useful in revealing biological changes, it is less sensitive than quantitative molecular methods. Furthermore, the absence of advanced molecular-level analyses in this study limits the detailed explanation of the mechanisms underlying the observed changes. The application methods of Ora-Aid and cyanoacrylate show no complete quantitative equivalence due to the physical properties of the materials; which means that the possible effect of material quantity on the healing process cannot be entirely excluded. Moreover, the retention of the wound dressings on the wound surface, local adhesion duration, and biocompatibility properties were not examined as separate evaluation parameters. Finally, although the obtained findings can be translated into clinical practice, further research involving longer follow-up periods, quantitative analyses, and human studies is needed to validate this potential.

## Conclusion

This study showed that Ora-Aid and cyanoacrylate affect oral mucosal wound healing differently depending on systemic condition, time point, and biological marker. Ora-Aid was observed to more prominently support VEGF-related angiogenic response under healthy conditions, and FGF-related fibroblastic activity under diabetic conditions. Cyanoacrylate, on the other hand, was found to exert time-dependent regulatory effects on growth factors and inflammatory response. Both materials showed supportive potential, particularly in delayed oral mucosal wound healing associated with diabetes.

## Supplementary Figure

Supplementary Figure 1Supplementary Figure 1 supporting the manuscript.
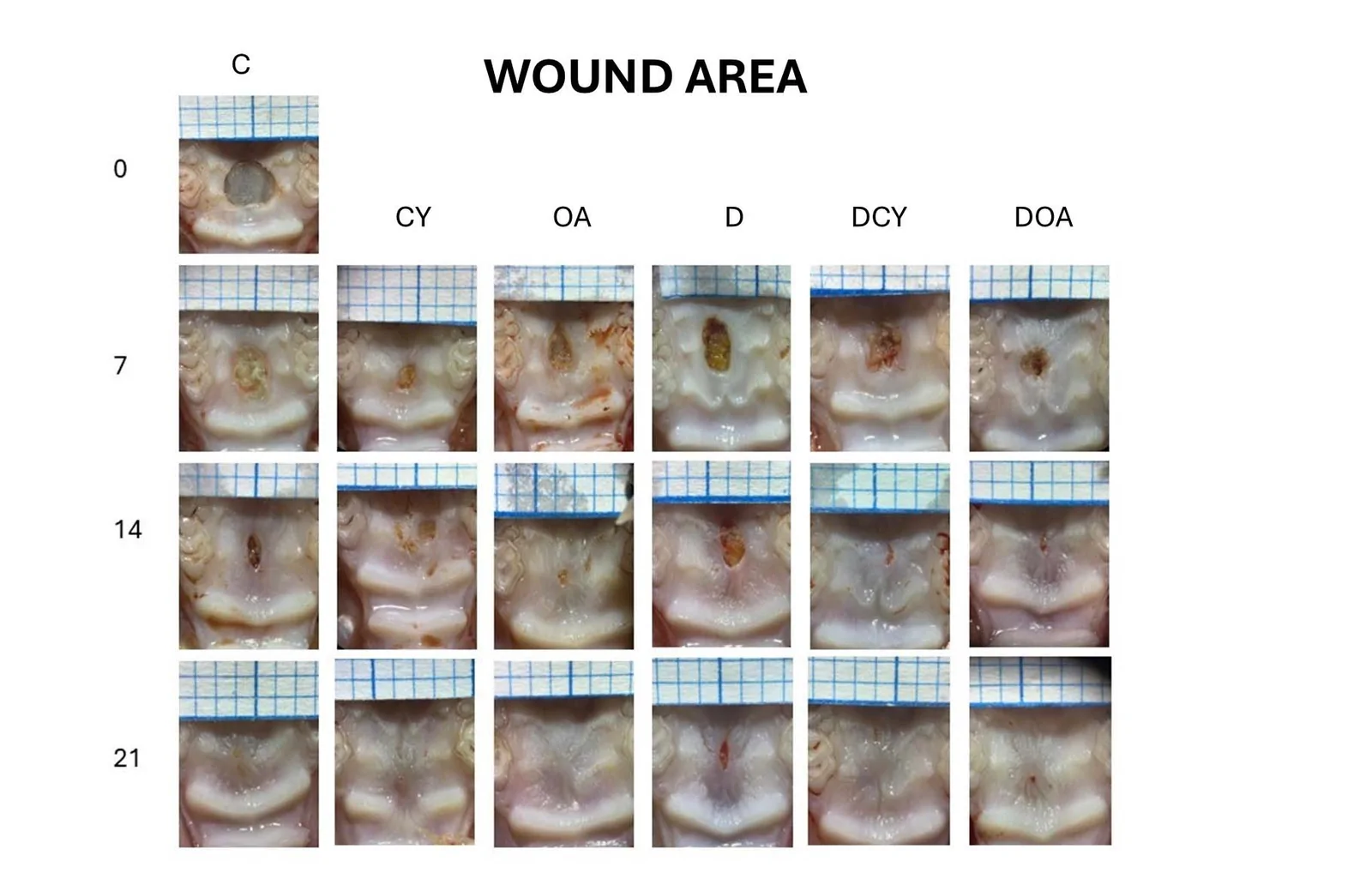
In Replication Data for: Comparison of the Effects of Two Different Wound Dressing Materials on Wound Healing in Mucosal Defects Created in Diabetic and Healthy Rats (version 1.0), by Yalçin, Merve; Bostanci, Vildan; Karataş, Özhan
